# Clamoring for Quiet: New Ways to Mitigate Noise

**DOI:** 10.1289/ehp.113-a46

**Published:** 2005-01

**Authors:** John Manuel

On a typical day in an American suburb, the steady whoosh of traffic on a nearby freeway drowns out the rustling of leaves in the wind. From across the street comes the nagging whine of a leaf blower, accompanied every few minutes by the deeper roar of a jet taking off from the airport. The cacophony of noise in the modern world is annoying to many and literally enough to make some people sick. Fortunately, new technologies are emerging to combat noise pollution.

## Quieter Airports Take Off

Sound levels are typically measured in decibels (dB). Humans hear sound within a limited frequency range, which is reflected in a value known as A-weighted dB, or dBA. According to community noise guidelines published in 1999 by the World Health Organization, for a good night’s sleep background sound levels should not exceed 30 dBA. In outdoor living areas, sounds above 50 dBA are annoying to humans. The Occupational Safety and Health Administration (OSHA) requires employers to provide workers with hearing protection if they are exposed to an 8-hour time-weighted average of 85 dBA or more. For those living or working near flight paths of major airports, the noise of aircraft taking off and landing can exceed 100 dBA.

Seven years after the passage of the National Environmental Policy Act of 1969, the Federal Aviation Administration (FAA) adopted the Aviation Noise Abatement Policy (ANAP), which among other things, sought to reduce aircraft noise at the source—the aircraft itself. Under ANAP, airlines have retired or replaced noisier aircraft in three stages. But while aircraft are now significantly quieter than they were a few decades ago, many airports have added new runways and increased the number of takeoffs and landings. And urban sprawl has resulted in more people living around airports than ever before. The result is continued public pressure to reduce aircraft noise.

The National Aeronautics and Space Administration (NASA) is spearheading research in reducing aircraft noise through its Quiet Aircraft Technology program. The FAA standard for aircraft noise is the EPNdB (or effective perceived noise dB—a measure that is weighted to reflect the particular range of sounds generated by aircraft). NASA aims to develop the technology to reduce commercial aircraft noise by 10 EPNdB by 2007 and another 10 EPNdB by 2019.

“Our goal is to provide the technology to contain all annoying aircraft noise within the airport boundary,” says Dennis Huff, chief of the Acoustics Branch at NASA’s Glenn Research Center in Cleveland, Ohio. “It will be up to regulations and the marketplace to decide when the technology is used before the noise reduction benefit is realized.”

Jet engine noise comes predominately from two sources. An approaching jet creates a high-pitched whine as the fan pulls air into the engine. As the jet passes by, a low-pitched rumble is created by exhaust leaving the engine.

Working with the major aircraft engine manufacturers, NASA has been able to reduce the former sound by designing engines with larger fans. Larger fan blades turn at a slower tip speed, which reduces both noise and fuel consumption. The turbo fan engines introduced in the 1970s are much quieter than the turbo jet engines they replaced, and engines being designed today are quieter still.

Different approaches are being used to reduce the noise produced by exhaust leaving the engine. Researchers have found that notching chevrons into the rim of the nozzle allows hot engine air to mix more thoroughly with the cooler ambient air. This decreases turbulence and reduces engine noise. Chevrons have been used so far on aircraft flown by America West and USAir. New engines with larger fans also slow exhaust air speed, for even more noise reduction.

## On the Road to Quieter Highways

Freeways are a ubiquitous source of noise pollution in urban America. Currently, barrier walls and earthen berms are the primary noise mitigation strategies, cutting the sound that reaches nearby homes by 10–15 dBA. However, these structures are expensive to build (often $1–2 million per mile) and to maintain (graffiti is a major problem). In addition, because sound waves have a tendency to bend over and around objects and to spread out with distance, barrier walls are only effective in reducing sound at distances of less than 400 meters from the roadway.

One of the more promising approaches to reducing road noise involves the use of rubberized asphalt pavement. In the late 1990s, the state of Arizona resurfaced a portion of Interstate 17 through central Phoenix using an asphalt rubber friction course (ARFC) overlay. Though the Arizona Department of Transportation (ADOT) had used this mix simply to extend the life of the concrete base, the public was more enthusiastic about how quiet it made the road.

Studies in Europe and Arizona have shown that resurfacing with ARFC can achieve noise level reductions of 3–5 dBA when compared to traditional asphalt dense-graded surfaces, and 6–12 dBA compared to concrete surfaces. As a tire passes over pavement, it causes a change in air pressure between the tire and the pavement, which generates sound. ARFC has many air pockets that dampen the air pressure gradient and thus reduce sound. In addition, the ARFC surface provides a smoother ride than concrete because it is laid in a continuous manner with minimal joints and a smaller aggregate (rock) mixed in.

In 2002, the Maricopa (Arizona) Association of Governments, the Phoenix area Metropolitan Planning Organization, and ADOT launched the $34 million Quiet Pavement Pilot Program, which so far has resurfaced more than 100 miles of highway with ARFC, says ADOT communications specialist Allison Saxe. ADOT is working with the Federal Highway Administration to collect sound measurements before and after the resurfacing to determine ARFC’s noise-reducing properties over time. So far the new pavement has yielded an average reduction of 4 dBA. Currently, the use of alternative pavement surfaces to achieve noise reduction is not eligible for federal funds unless the state can provide data proving the noise-reduction properties of the material and makes a commitment to repave or erect sound barriers if noise reduction diminishes over time.

## Constructing Quieter Buildings

Manufacturers of building components are also making exciting advances in the field of noise reduction. Traditionally, architects and builders have used two methods to reduce sound transmission through walls, floors, and ceilings. The first is to install materials with air pockets (e.g., insulation) that trap sound waves; the second is to increase wall thickness. These approaches may work for new construction, but they are difficult and costly to implement in existing buildings, where walls must be gutted and rebuilt. Recently, Quiet Solution, a California-based manufacturer of soundproofing materials, introduced a product line that can easily be added to new or existing walls to achieve remarkable reductions in sound transmission.

Quiet Solution’s breakthrough product line of drywalls, caulks, tiles, and other materials employs a viscoelastic polymer that converts sound waves to harmless heat. The polymer kills vibration and is more effective with successive layers applied. The ability of building materials to reduce sound transmission is typically classified according to “sound transmission class,” or STC. Typical interior wall construction using wood studs sheathed in drywall has an STC rating of 30–34. By adding a 5/8”-thick sheet of Quiet Solution drywall to both sides of an existing wall, the STC rating jumps to 56, which translates into an 86% perceived sound reduction. In new construction, it is possible to obtain an STC rating of 70 using two layers of wood studs and a layer of Quiet Solution drywall on each side.

Marc Porat, chairman and founder of Quiet Solution, explains what this might mean in today’s home environment. “A typical home theater produces sounds as loud as a hundred decibels. A room built with standard-construction walls adjacent to the home theater would have sound levels of about seventy decibels, which is far too loud for conversation. However, with a wall built to an STC of sixty, the adjacent room would have sound levels of forty decibels, about as quiet as a library.”

## Active Noise Control

Another noise reduction technology that is making significant inroads in certain sectors employs the concept of “active noise control” (ANC). In its simplest form, ANC involves producing a sound field that is the mirror image of the offending sound. In essence, active noise cancels out the disturbance, with the net result that the sound is significantly reduced. A sensor such as a microphone, accelerometer, or other device picks up the annoying sound and relays the signal to an electronic controller, which drives an actuator (an electromagnetic speaker or vibration generator) to generate the opposing sound.

ANC works best for controlling narrow-bandwidth, low-frequency sounds, such as air traveling through a duct. The technology is enjoying widespread use in the industrial sector for silencing the noise of large industrial fans, compressors, and generators.

“There are half a dozen companies around the world installing active noise control technologies in industry,” says Rich Silcox, assistant head of NASA’s Structural Acoustics Branch at Langley Research Center. “Most of these devices have to be tailored to specific applications. There are not a lot of off-the-shelf products.”

One consumer product that does employ ANC is headphones available through specialty mail-order catalogues and retailers such as Brookstone and Bose. ANC headphones typically cost several hundred dollars. They are used extensively by pilots and airport workers, but are becoming increasingly popular with the public to ensure quiet airplane rides and work environments.

A related approach to noise control is called active structural–acoustic control (ASAC). In this system, the actuators are vibration sources such as shakers or piezoceramic patches, which use an electric current to introduce an additional vibration that is out of phase with the vibration of the surface they are attached to. These actuators can modify how a structure vibrates, thereby altering the way it radiates sound. This type of system is used in helicopters, for example, to counter noise generated by the rotor and gear box. Some turboprop aircraft employ a combination of ANC and ASAC devices to counter the sound and vibration generated by the propellers.

## Mowing Down Noise

One of the most pervasive sources of noise pollution in suburban neighborhoods is lawn equipment. The Noise Pollution Clearinghouse (NPC), a nonprofit agency providing access to a variety of materials on controlling noise pollution, has chosen lawn equipment as one of its primary focuses.

“The average life of the lawn mowers and weed trimmers in the United States today is about seven years,” says NPC executive director Les Blomberg. “By 2011 most of today’s stock will be in the recycle heap. There is a tremendous opportunity to reshape our neighborhood soundscapes by reshaping the lawn and garden marketplace.”

According to data provided by the NPC’s annual “Quiet Lawns” report, which rates various brands of lawn mowers on noisiness, a typical two-stroke gas-powered lawn mower subjects the operator to 85–90 dBA and should be operated only while wearing hearing protection. The latest (2004 model year) gas mowers employ four-stroke engines producing as little as 82 dBA. Electric-powered lawn mowers are quieter still, with the best model emitting only 68 dBA and not requiring the use of hearing protection. For small, evenly contoured lawns, consumers may want to purchase an old-fashioned reel lawn mower, used by golf courses because of their better cut. Some models produce as little as 63 dBA.

The NPC will be adding ratings for weed trimmers and chain saws to its annual report. “Our motto is ‘good neighbors keep their noise to themselves,’” Blomberg says.

## Seeking Silence

As awareness is raised about the effects of noise on human health and well-being, public demand for controlling that noise will increase. In the not-too-distant future, technologies for developing machines that generate excessive sound may also incorporate the technology to suppress it. For societies seeking to cope with sensory overload, devices and innovations to reduce the sounds of modern life—and thus noise pollution—are good news indeed.

## Figures and Tables

**Figure:**
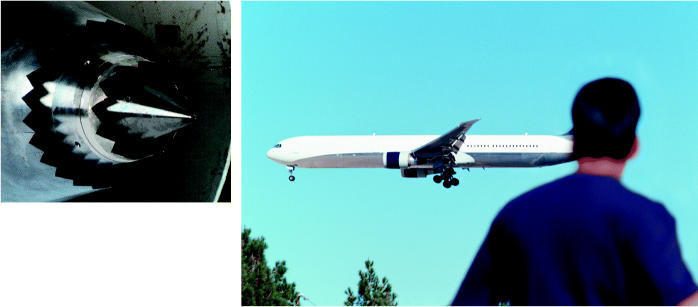
**Just plane brilliant?** Chevron nozzles (above) reduce the sound of exiting jet engine exhaust.

**Figure:**
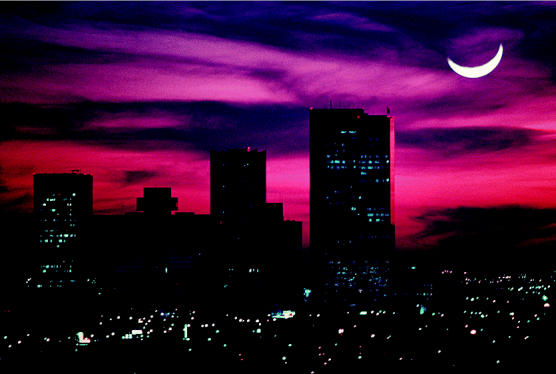
**Asphalt for a better sleep.** Rubber-augmented pavement cuts down on road noise in Phoenix, Arizona.

**Figure:**
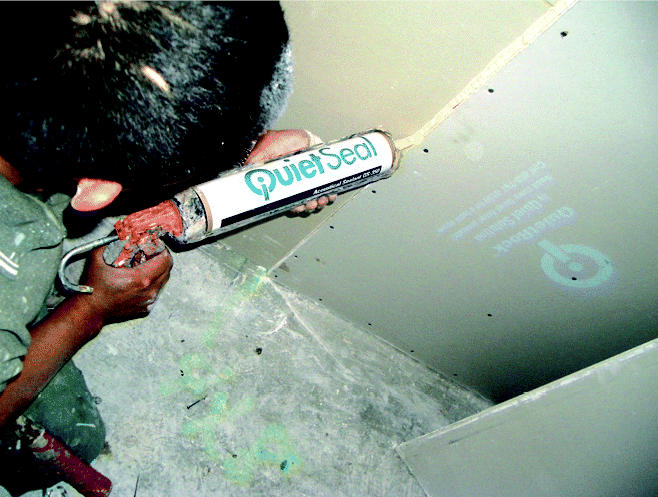
**Caulk for quieter walls.** New drywall and caulk are engineered to muffle building noise.

**Figure:**
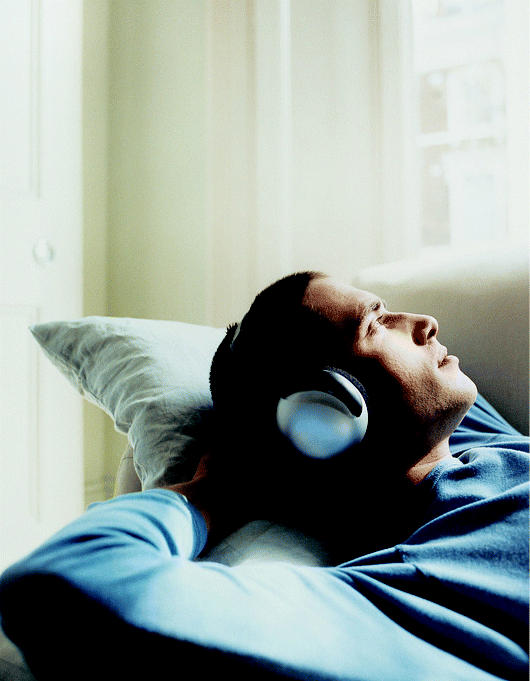
**Sounding out quiet.** New head phones use active noise control to counter unwanted sounds.

**Figure:**
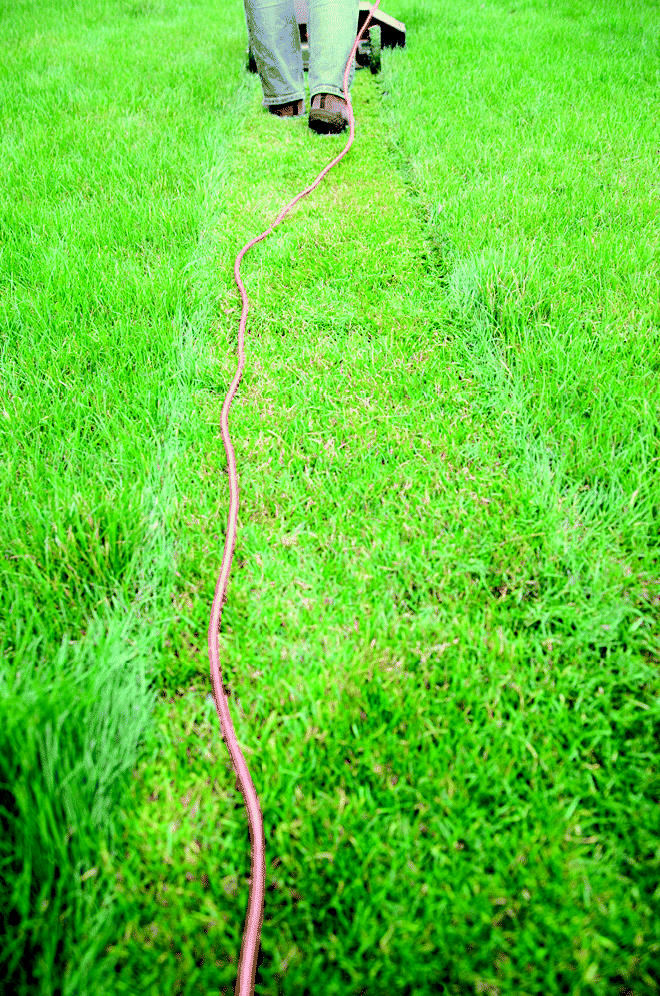
**Cutting with cords.** Electric lawn mowers can produce sound levels as low as 68 dBA.
